# Supplementary material for: Cryo-EM structures of apo-APC/C and APC/CCDH1:EMI1 complexes provide insights into APC/C regulation
Source: Nat Commun. 2024 Nov 21;15:10074. doi: 10.1038/s41467-024-54398-5 (PMC11579458; doi:10.1038/s41467-024-54398-5)
Supplement: Supplementary file 1 — Supplementary Information [file 41467_2024_54398_MOESM1_ESM.pdf]

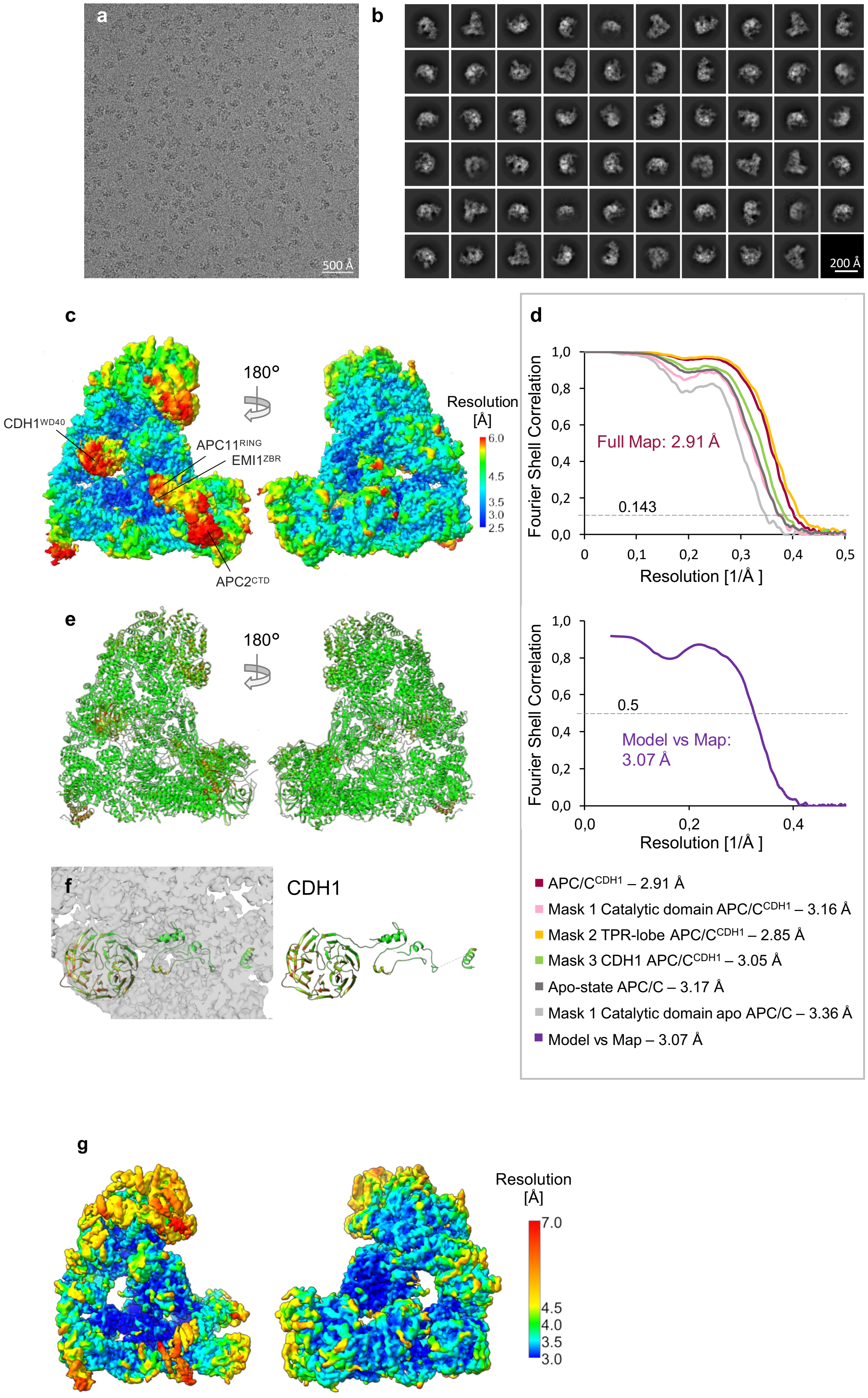

a
500 Å
b
200 Å
c
180°
CDH1WD40
APC11RING
EMI1ZBR
APC2CTD
Resolution [Å]
6.0
4.5
3.5
3.0
2.5
d
Fourier Shell Correlation
Full Map: 2.91 Å
0.143
Resolution [1/Å]
Model vs Map: 3.07 Å
0.5
e
180°
f
CDH1
APC/CCDH1 – 2.91 Å
Mask 1 Catalytic domain APC/CCDH1 – 3.16 Å
Mask 2 TPR-lobe APC/CCDH1 – 2.85 Å
Mask 3 CDH1 APC/CCDH1 – 3.05 Å
Apo-state APC/C – 3.17 Å
Mask 1 Catalytic domain apo APC/C – 3.36 Å
Model vs Map – 3.07 Å
g
Resolution [Å]
7.0
4.5
4.0
3.5
3.0

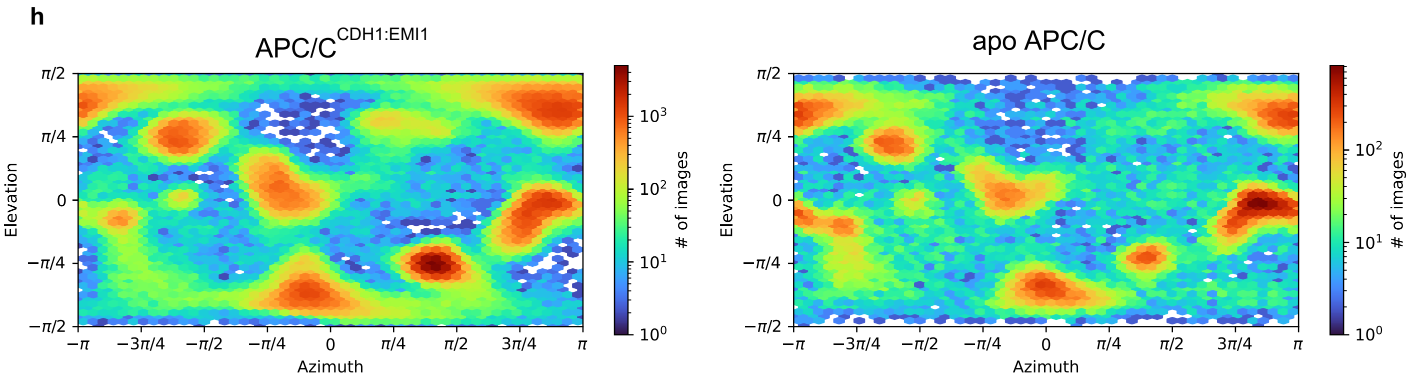

Supplementary Figure 1. Cryo-EM images and refined APC/C reconstructions. a, Representative cryo-EM image of the APC/C$^{CDH1:EMI1}$ complex (from a total of 8,296 images). **b**, Gallery of 2D-class averages of APC/C$^{CDH1:EMI1}$. **c**, Local resolution map of APC/C$^{CDH1:EMI1}$. **d**, Fourier Shell Correlation (FSC) plots of the main maps used to generate molecular models. **e**, Q-score analysis [1] of the APC/C$^{CDH1:EMI1}$ complex to validate map-model correlation. **f**, Close-up view on the CDH1 subunit of the APC/C$^{CDH1:EMI1}$ complex that is colour-coded by Q-score. **g**, Local resolution map of apo APC/C. **h**, Plots of angular distribution of particles used in final reconstructions: left panel – APC/C$^{CDH1:EMI1}$; right panel – apo APC/C.

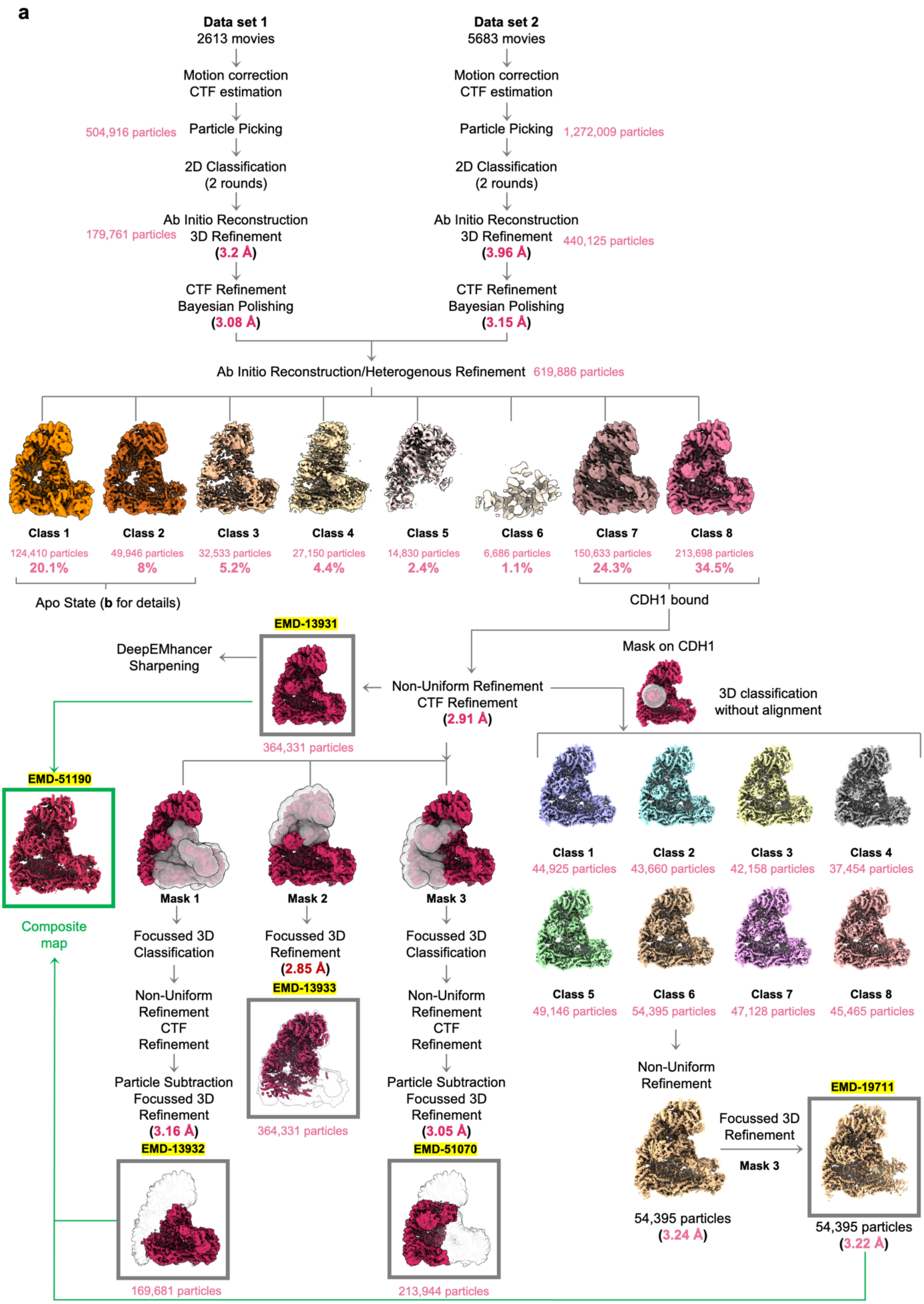

a
Data set 1
2613 movies
Motion correction
CTF estimation
504,916 particles
Particle Picking
2D Classification
(2 rounds)
Ab Initio Reconstruction
179,761 particles
3D Refinement
(3.2 Å)
CTF Refinement
Bayesian Polishing
(3.08 Å)
Data set 2
5683 movies
Motion correction
CTF estimation
Particle Picking
1,272,009 particles
2D Classification
(2 rounds)
Ab Initio Reconstruction
3D Refinement
440,125 particles
(3.96 Å)
CTF Refinement
Bayesian Polishing
(3.15 Å)
Ab Initio Reconstruction/Heterogenous Refinement
619,886 particles
Class 1
124,410 particles
20.1%
Class 2
49,946 particles
8%
Class 3
32,533 particles
5.2%
Class 4
27,150 particles
4.4%
Class 5
14,830 particles
2.4%
Class 6
6,686 particles
1.1%
Class 7
150,633 particles
24.3%
Class 8
213,698 particles
34.5%
Apo State (b for details)
CDH1 bound
EMD-13931
DeepEMhancer
Sharpening
Mask on CDH1
Non-Uniform Refinement
CTF Refinement
(2.91 Å)
3D classification
without alignment
364,331 particles
EMD-51190
Composite
map
Mask 1
Focussed 3D
Classification
Non-Uniform
Refinement
CTF
Refinement
Particle Subtraction
Focussed 3D
Refinement
(3.16 Å)
EMD-13932
169,681 particles
Mask 2
Focussed 3D
Refinement
(2.85 Å)
EMD-13933
364,331 particles
Mask 3
Focussed 3D
Classification
Non-Uniform
Refinement
CTF
Refinement
Particle Subtraction
Focussed 3D
Refinement
(3.05 Å)
EMD-51070
213,944 particles
Class 1
44,925 particles
Class 2
43,660 particles
Class 3
42,158 particles
Class 4
37,454 particles
Class 5
49,146 particles
Class 6
54,395 particles
Class 7
47,128 particles
Class 8
45,465 particles
Non-Uniform
Refinement
54,395 particles
(3.24 Å)
Focussed 3D
Refinement
Mask 3
EMD-19711
54,395 particles
(3.22 Å)

b

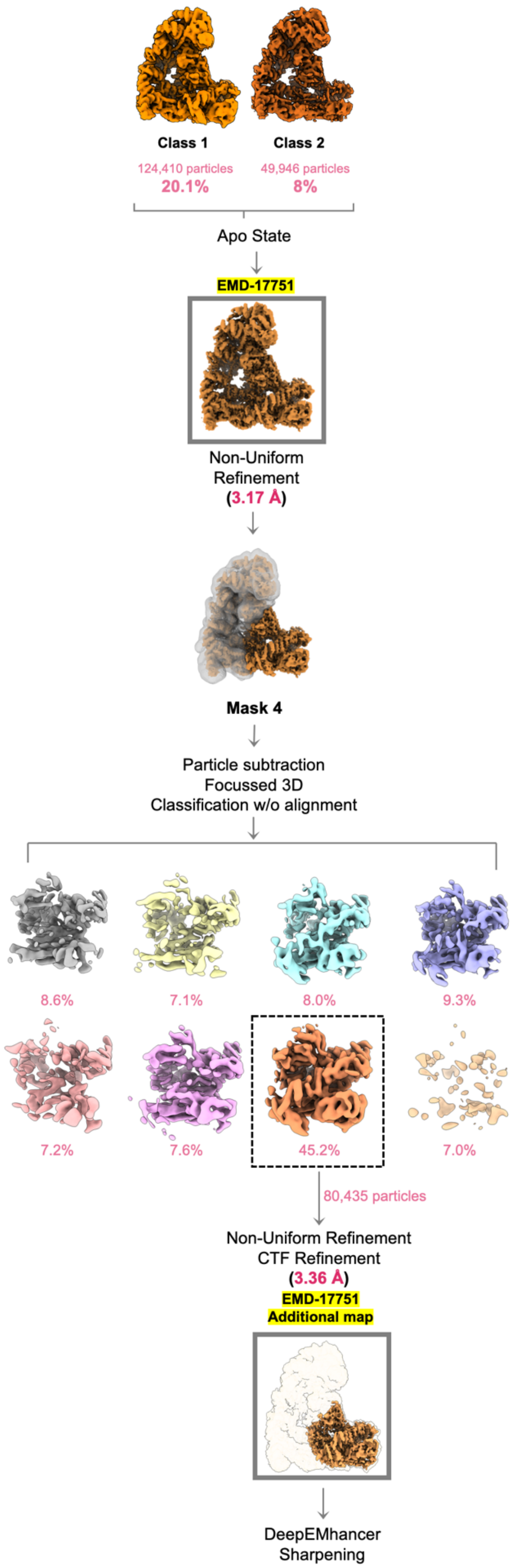

Supplementary Figure 2. Data processing pipeline for cryo-EM reconstructions. (a) Cryo-EM data processing workflow summary as described in the Methods section for the ternary APC/C$^{CDH1:EMI}$ complex. (**b**) Work flow for apo-APC/C.

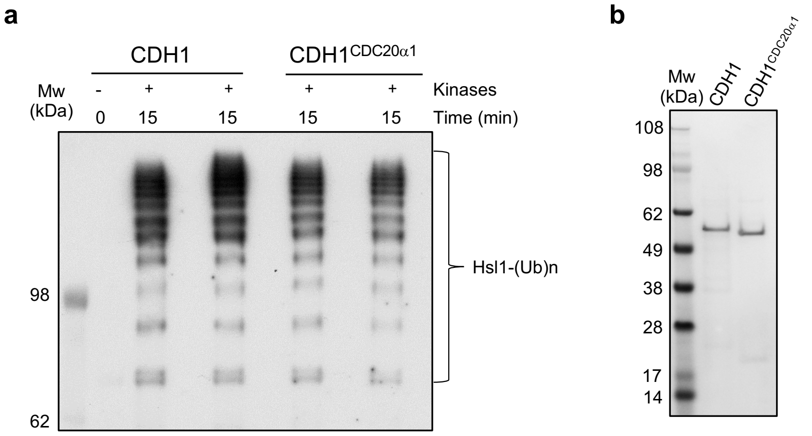

Supplementary Figure 3. CDH1 and CDH1$^{CDC20\alpha1}$ activate unphosphorylated and phosphorylated APC/C to similar extents. a, Ubiquitination assay with unphosphorylated and phosphorylated APC/C activated by CDH1 and CDH1$^{CDC20\alpha1}$. **b**, SDS PAGE gel of CDH1 and CDH1$^{CDC20\alpha1}$ used in the ubiquitination assay. These experiments were performed in triplicate. Source data are provided as a Source Data file.

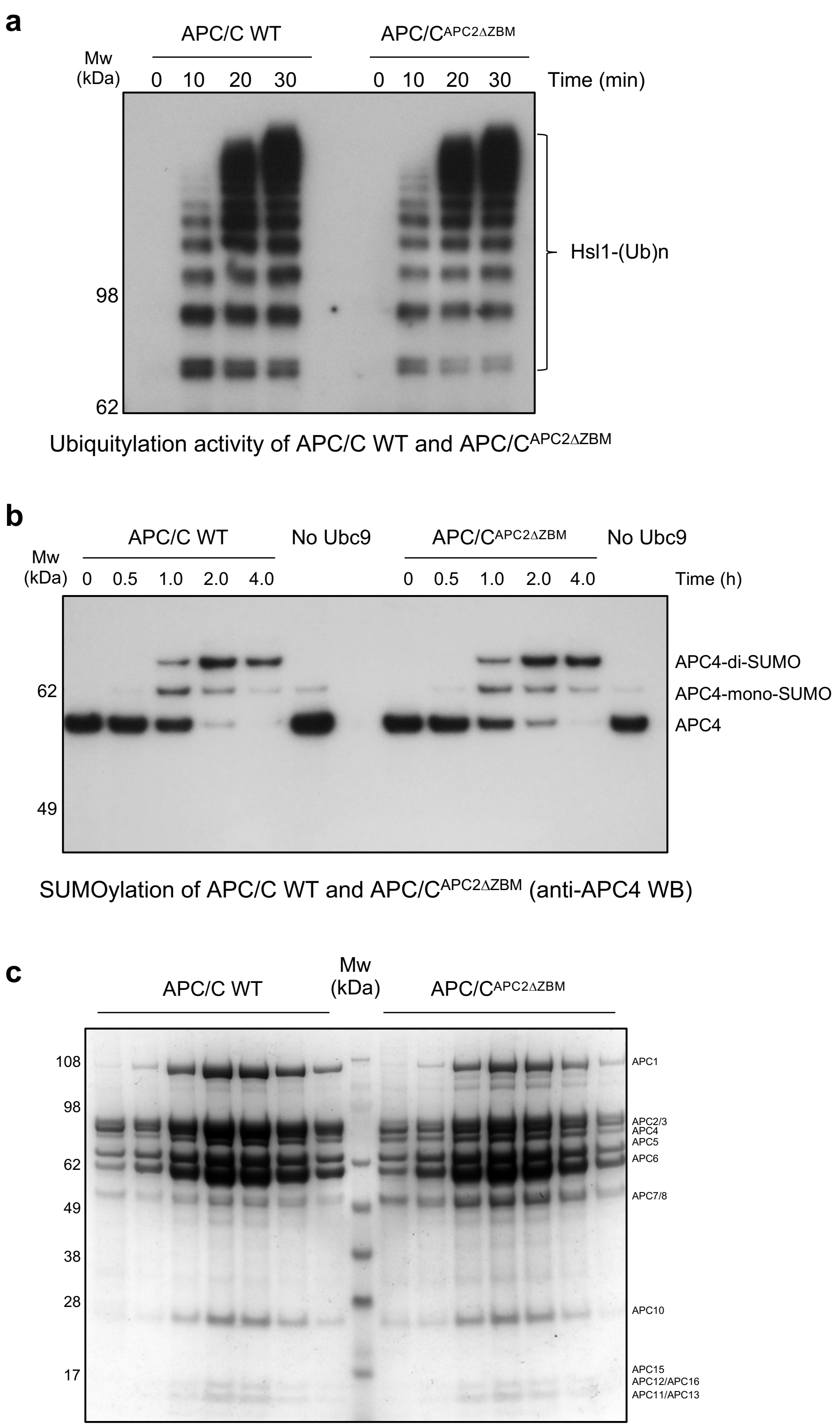

Supplementary Figure 4. Wild type APC/C and the APC/C$^{APC2\Delta ZBM}$ mutant have similar ubiquitination activity and are SUMOylated to the same extent. a, A ubiquitylation assay shows wild type APC/C and the APC/C$^{APC2\Delta ZBM}$ mutant (disrupted APC2^{ZBM}) are equally active as E3 ligases. **b**, SUMOylation assays shows that wild type APC/C and the APC/C$^{APC2\Delta ZBM}$ mutant are SUMOylated to the same extent. APC4 SUMOylation is shown by a Western blot against APC4. **c**, SDS PAGE gel comparing wild type APC/C and mutant APC/C$^{APC2\Delta ZBM}$. The mutant APC2$^{\Delta ZBM}$ did not impair APC/C assembly. These experiments were performed in triplicate. Source data are provided as a Source Data file.

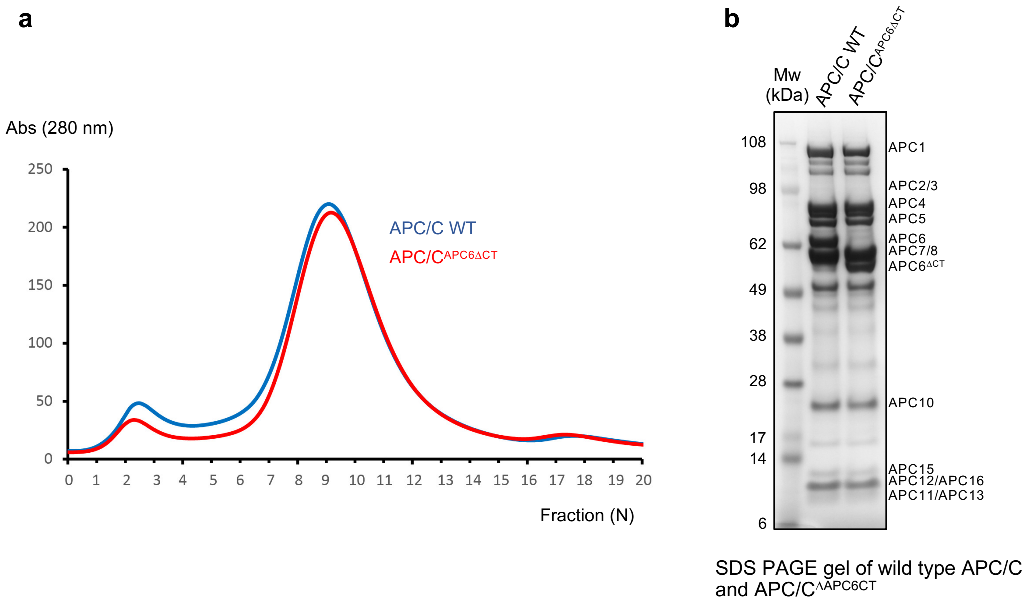

Supplementary Figure 5. APC/C$^{APC6\Delta CT}$ assembles normally. a, Size exclusion chromatogram and **b**, SDS PAGE gel of wild type APC/C and APC/C$^{APC6\Delta CT}$ show similar elution profiles on a SEC column (a), and subunit stoichiometry (b). These experiments were performed in triplicate. Source data are provided as a Source Data file.

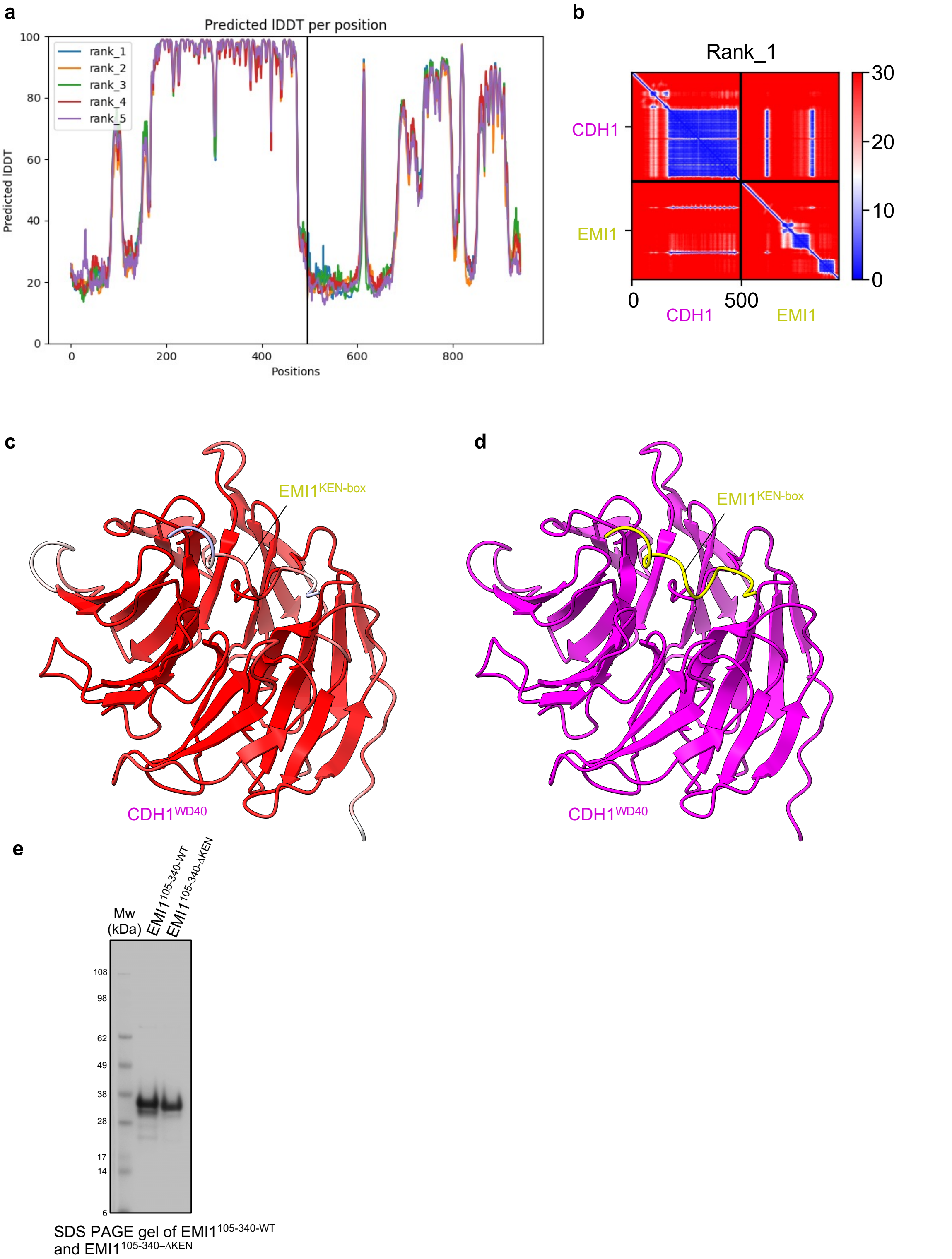

Supplementary Figure 6. AlphaFold2 prediction of the CDH1^{WD40}:EMI1$^{KEN\ box}$ interaction and confidence metrics. **a**, Predicted local distance difference test (lDDT) values for the AlphaFold2 models of CDH1^{WD40}:EMI1$^{KEN\ box}$. A higher score indicates higher confidence in the prediction. **b**, The predicted alignment error (PAE) heat map for the CDH1^{WD40}:EMI1$^{KEN\ box}$ AlphaFold2 prediction. The PAE heat map shows the predicted error (in angstroms) between all pairs of residues, with blue indicating lower error and red indicating higher error. The PAE plot suggests high confidence for the predicted interactions between CDH1^{WD40} and EMI1$^{KEN\ box}$. **c**, The pLDDT values mapped onto the predicted model of CDH1^{WD40}:EMI1$^{KEN\ box}$. Residues are coloured in a scale of high (red) to low (blue) pLDDT values. The model was predicted with high confidence (pLDDT > 90). **d**, Predicted model colour-coded according to Fig. 1c. **e**, SDS PAGE gel of EMI1$^{105\text{-}340\text{-}WT}$ and EMI1$^{105\text{-}340\text{-}\Delta KEN}$. This experiment was performed in triplicate. Source data are provided as a Source Data file.

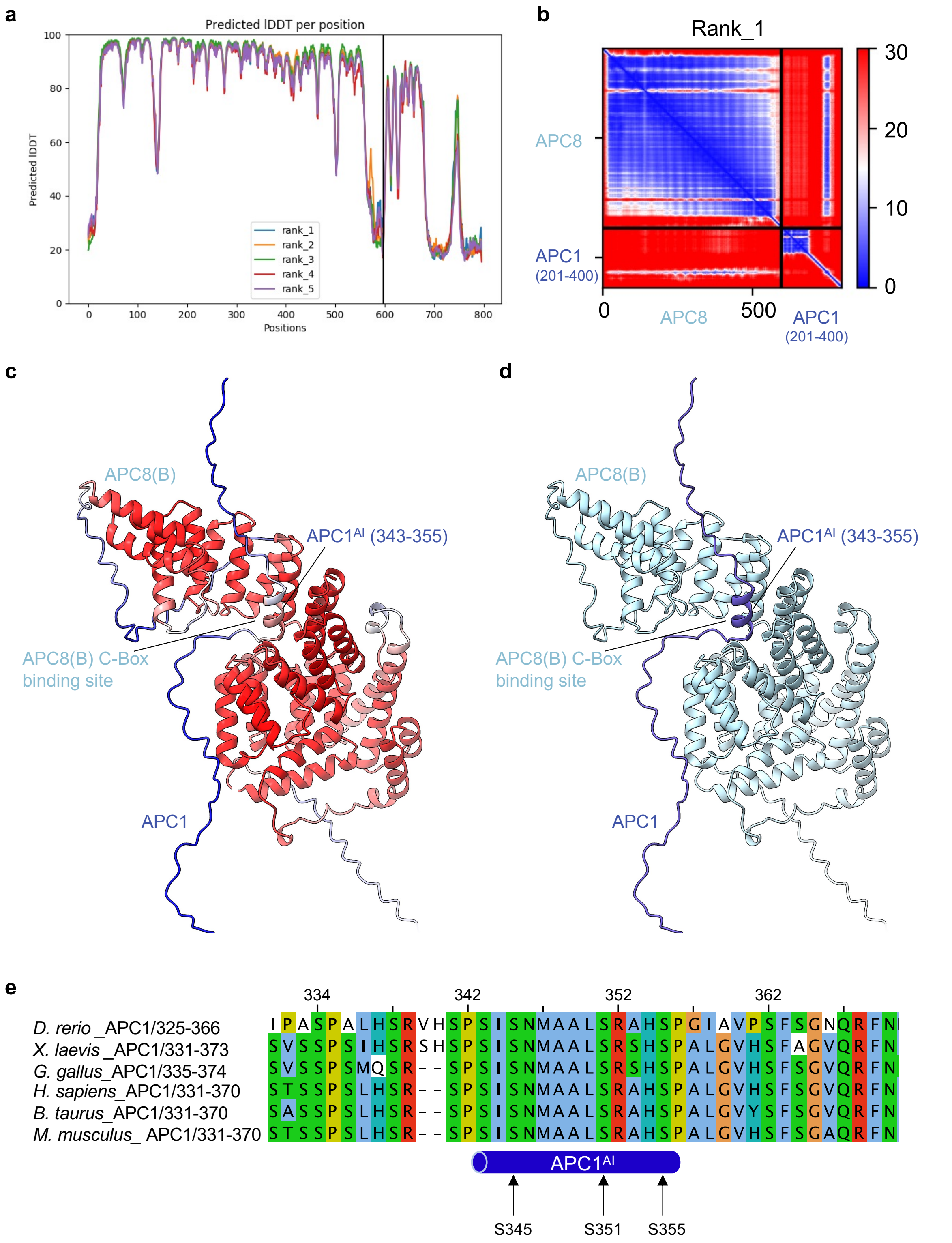

Supplementary Figure 7. AlphaFold2 prediction of the APC1[AI]:APC8 interaction and confidence metrics. **a**, Predicted local distance difference test (lDDT) values for the AlphaFold2 models of APC1[AI]:APC8. A higher score indicates higher confidence in the prediction. **b**, The predicted alignment error (PAE) heat map for the APC1[AI]:APC8 AlphaFold2 prediction. The PAE heat map shows the predicted error (in angstroms) between all pairs of residues, with blue indicating lower error and red indicating higher error. The PAE plot suggests high confidence for the predicted interactions between APC1[AI] and APC8. **c**, The pLDDT values mapped onto the predicted model of APC1[AI]:APC8. Residues are coloured in a scale of high (red) to low (blue) pLDDT values. The model was predicted with high confidence (pLDDT > 90). **d**, Predicted model colour-coded according to Fig. 1c. **e**, MSA of APC1 comprising the AI segment of selected vertebrate species. Sites of phosphoryation are indicated (Ser345, Ser351, Ser355).

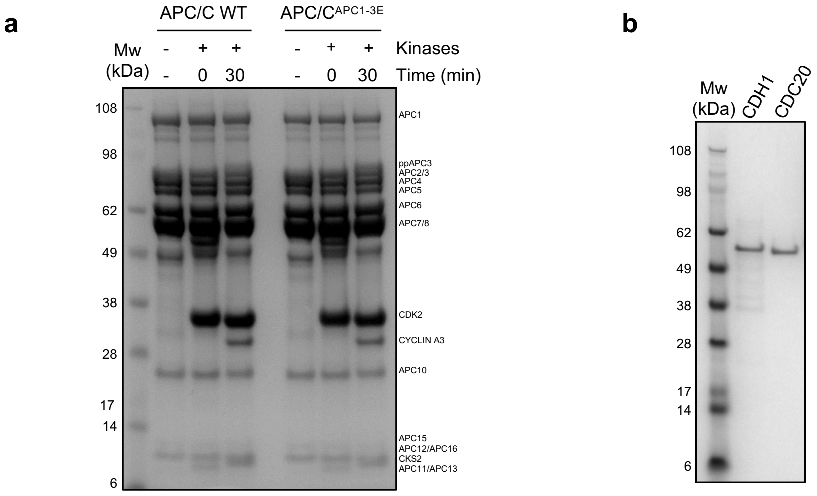

SDS PAGE gel of wild type APC/C and $APC/C^{APC1-3E}$ before and after phosphorylation

SDS PAGE gel of CDH1 and CDC20

Supplementary Figure 8. SDS PAGE gels of purified APC/C complexes and coactivator. **a**, SDS PAGE gel of unphosphorylated and phosphorylated wild type APC/C and $APC/C^{APC1-3E}$ mutant. The slower migrating phosphorylated APC3 subunit (ppAPC3) indicates APC/C phosphorylation. **b**, SDS PAGE gel of CDH1 and CDC20 used in this assay. These experiments were performed in triplicate. Source data are provided as a Source Data file.

Supplementary Table 1. Cryo-EM data collection, refinement and validation statistics

		APC/C$^{CDH1:EMI1}$ Dataset 1			APC/C$^{CDH1:EMI1}$ Dataset 2		
**Data collection**							
Microscope		FEI Titan Krios			FEI Titan Krios		
Voltage (keV)		300			300		
Electron dose (e^-/Å^{-2})		40			40		
Detector		FEI Falcon III			FEI Falcon III		
Pixel size (Å/pixel)		1.07			1.07		
Defocus range (μm)		1.5-3.5			1.5-3.5		
Micrographs (N)		2,613			5,683		
**Reconstructions**							
	APC/C^{apo}	APC/C$^{CDH1:EMI1}$ Composite map	APC/C$^{CDH1:EMI1}$ Consensus map	APC/C$^{CDH1:EMI1}$ Focussed Map Catalytic domain	APC/C$^{CDH1:EMI1}$ Focussed Map TPR lobe	APC/C$^{CDH1:EMI1}$ Focussed Map CDH1	APC/C$^{CDH1:EMI1}$ Improved CDH1 occupancy
				Mask1	Mask2	Mask3	
Particles (N)	174,356	-	364,331	169,681	364,331	213,944	54,395
Box size (pix)	360	360	360	360	360	360	360
Map sharpening				-		-	
B-factor (Å^2)	-116.8	-	-119.1	106.3	-117.3	121.3	-118.5
Resolution (global, Å)	3.2	-	2.9	3.16	2.85	3.05	3.24
Resolution range (local, Å)	2.7-9.1	-	2.4-7.8	2.6-9.0	2.4-8.5	2.6-8.6	2.7-9.5
FSC threshold	0.143	-	0.143	0.143	0.143	0.143	0.143
**Model composition**							
Protein residues (N)	8,076	8,600					
Zn ions (N)	4	6					
**Refinement**							
Resolution (Å)	3.4	3.3					
FSC threshold	0.5	0.5					
Model to map (CC)	0.78	0.81					
B factors (Å^2)							
Protein residues	-79.1	-62.9					
Ligands	-205.5	-184.5					
RMS deviation							
Bond lengths (Å)	0.004	0.005					
Bong angles (°)	0.642	1.008					
**Validation**							
Clashscore, all atoms	12	11					
Rotamer outliers (%)	2.3	0.8					
**Ramachandran plot**							
Favoured (%)	97.4	96.9					
Allowed (%)	2.60	3.11					
Outliers (%)	0	0.02					
**Deposition**							
PDB ID	8PKP	9GAW					
EMDB ID	EMD-17751	EMD-51190	EMD-13931	EMD-13932	EMD-13933	EMD-51070	EMD-19711

Supplementary Table 2. Rebuilt regions of APC/C$^{CDH1:EMI1}$ subunits PDB: 9GAW

Subunit	Residue range	Modification	Comments	4ul9
APC1	135-145	Loop refitted		
	192-206	Loop refitted		
	275-283	Rebuild		
	282-295	α-helix to loop<br>Helix now Cdh1^N		
	415-424	Loop fitted		
	460-464	Loop fitted		
	646-650	Loop rebuilt		
	671-681	α-helix built		
	701-734	Build		SC unassigned
	741-757	Build		
	814-838	Build		
	853-859	Build		
	882-901	Build		
	1009-1013	Build		
	1333-1347	Build		
	1433-1452	Build		
	1673-1684	Rebuild		
	1711-1734	Rebuild/build		
	1734-1911	Renumber -3		
	1825-1839	Build		
	1873-1877	Build		
	1897-1911	Build		
	1897-1936	Rebuild		
	1911-1935	Reverse chain polarity, fit s.c.		
APC2	17-35	s.c. built to α-helix		15-28, m.c. only
	46-52	Build		
	53-87	Rebuild and fit s.c.		
	126-147	Fit s.c.		Poly A
	187-233	Build/rebuild, fit s.c.	New Zn-binding module built: Cys 221,224, 231,233	
	304-322	Loop fitted		
	458-507	Rebuild		
	605-640 (CTD)		Good density	
	644-659 (CTD)		β-hairpin no density	
	660-716 (CTD)	m.c. good, s.c. poorly defined	Only poor part of APC2	
APC3(B) chain P	171-176	Build		
	177-445		Long disordered region	
	446-450	Build		
APC3(A) chain J	2-4	Build		
	171-176	Build		
	177-445		Long disordered region	
	446-450	Build		
	767-780	Rebuild		
APC4	126-133	Loop fitted		

	276-301	Rebuild		
	430-439	Loop fitted		
	465-470	Loop fitted		
	487-495	Rebuild		
APC5	9-27	Build		
	46-55	Rebuild		
	351-357	Rebuild		
	742-755	Rebuild		
APC6(A) chain Q	No change			
APC6(B) chain K	94-98	Build		
	120-128	Build		
	544-567	Build: fit s.c. assigned to APC6		Poly A, unassigned chain
APC7(A) chain Y	34-35	Build		
	553-555	Build		
APC7(B) chain Z	34-35	Build		
	131	Build		
	111-122	Build		
	371-540	Reposition α-helices		
APC8(A) chain U	499-509	Loop fitted		
	504-523	Rebuild		
APC8(B) chain V	499-509	Loop fitted		
APC10	167-176	Rebuild, s.c. fitted		
APC11	1-8	Build β-strand		
	18-84	RING domain	m.c. and s.c.: good fit	
APC12(A) chain G	26-27	Build		
APC12(B) chain W	26	Build		
APC13	1-6	Rebuild		
	39-47	Build		
	68	Build		
APC15	No change			
APC16	51-57	Rebuild		
	108	Build		
CDH1	1-17	Build as α-helix		α-helix assigned to APC1 284-295
	144-145	Build		
	164-172	Build	Connects to WD40	
	473-476	Rebuild		
EMI1	117-121	Build	KEN-box	
		ZBR domain 356-415	m.c. and s.c.: good fit	
	433-447	Fit s.c.	Incudes LRRL	

Supplementary Table 3. Ordered and disordered regions of APC/C subunits

Subunit	Visible N-term	Visible C-term	Disordered regions	[6]AF2 C-term helix	Protein Length (N)
APC1	10	1936	51-70, 195-204, 228-233, 284-399 (AI segment), 516-581, 682-700, 735-741, 902-921, 987-1010, 1336-1346, 1439-1451, 1902-1907, 1937-1944		1944
APC2	17	716	35-48, 307-317, 461-474, 463-470, 488-495, 646-657, 707-711, 717-822 (WHB domain)		822
[1,2]APC3(A) chain J	3	780	172-450, 781-824	783	824
[1,2]APC3(B) chain P	5	769	174-447, 770-824	783	824
APC4	3	755	128-133, 431-437, 458-468, 756-808		808
APC5	9	755	20-25, 168-204 (linker), 453-457		755
[3,4]APC6(A) chain Q	1	533	96-123 (AF2 α-helix 99-109), 534-630	528	620
[3]APC6(B) chain K	1	565	99-126 (AF2 α-helix 99-109), 528-544, 566-620	528	620
APC7(A) chain Y	35	540	111-131, 541-565, AF2 α-helix 3-23 not visible	557	565
APC7(B) chain Z	35	507	111-130, 508-565, AF2 α-helix 3-23 not visible	557	565
[5]APC8(A) chain U	25	523	136-144, 524-59	559	597
[5]APC8(B) chain V	24	523	523-597	559	597
APC10	4	185	164-167		185
APC11	3	83	84		84
APC12(A) chain W	1	26	27-85		85
APC12(B) chain G	1	26	27-85		85
APC13	1	68	42-45, 69-74		74
APC15	2	57	58-121		121
APC16	51	108	1-50, 109-110		110
CDH1	1	496	14-41, 68-87, 110-122,134-142, 416-434		496
EMI1	116	447	1-115, 123-319, 335-357, 416-434		447

Notes

[1]Different trajectory N-term of IR tail for CDH1 and APC10
[2]Residues 273-286 of APC3 insert predicted to bind IR-tail binding site of APC3, includes $T^{279}P$
[3]AF2: In free APC6, residues 596-620 of APC6 mimic residues 1-27 of APC12
[4]Residues 529-540 of APC6(A) chain Q interacts with APC8(A) chain U
[5]AF2: Residues 587-C-term of APC8 binds to C-box/IR tail site of APC8
[6]C-terminal residue of an AF2-predicted α-helix.

N-term and C-term residues of sequence

Supplementary References

1 Pintilie, G. *et al.* Measurement of atom resolvability in cryo-EM maps with Q-scores. *Nature methods* **17**, 328-334, doi:10.1038/s41592-020-0731-1 (2020).
