# Supplementary material for: Cryo-EM structures of apo-APC/C and APC/CCDH1:EMI1 complexes provide insights into APC/C regulation
Source: Nat Commun. 2024 Nov 21;15:10074. doi: 10.1038/s41467-024-54398-5 (PMC11579458; doi:10.1038/s41467-024-54398-5)
Supplement: Supplementary file 2 — Description of Additional Supplementary Files [file 41467_2024_54398_MOESM2_ESM.pdf]

<u>Description of Additional Supplementary files</u>

Supplementary Movie 1: Video shows conformational change on transition from apo-APC/C to the APC/$C^{CDH1:EMI1}$ ternary complex. Apo-APC/C and APC/$C^{CDH1:EMI1}$ were superimposed on $APC1^{PC}$.
